# *Helicobacter pylori* infection activates Wnt/β-catenin pathway to promote the occurrence of gastritis by upregulating ASCL1 and AQP5

**DOI:** 10.1038/s41420-022-01026-0

**Published:** 2022-05-10

**Authors:** Wei Zuo, Hui Yang, Nianshuang Li, Yaobin Ouyang, Xinbo Xu, Junbo Hong

**Affiliations:** 1 Department of Respiratory Medicine, The First Affiliated Hospital of Nanchang University, Nanchang, 330006 P. R. China; 2 Department of Gastroenterology, The First Affiliated Hospital of Nanchang University, Nanchang, 330006 P. R. China

**Keywords:** Inflammasome, HIPPO signalling

## Abstract

*Helicobacter pylori (H. pylori)* infection is a well-recognized contributing factor to gastritis, but the underlying mechanisms remain to be established. It is interesting to note that AQP5 was predicted to be highly expressed in intestinal metaplasia (IM) based on *H. pylori* infection-related microarray data, and the transcription factor ASCL1 was bioinformatically predicted to associate with AQP5. Therefore, the purpose of this study is to evaluate the mechanistic significance of ASCL1 and AQP5 in *H. pylori* infection of gastritis. Gastritis mouse models were established by *H. pylori* infection, followed by determination of AQP5 and ASCL1 in gastric mucosa. Besides, the effects of AQP5 on *H. pylori*-induced gastritis were explored using AQP5^−/−^ mice. It was observed that *H. pylori* infection elevated expression of AQP5 and ASCL1 in gastric mucosa and gastric epithelial cells (GECs). *H. pylori* induced AQP5 expression by regulating ASCL1 and activated WNT/β-catenin signaling pathway in GECs. It was also found that AQP5 knockdown suppressed inflammatory response and apoptosis in *H. pylori*-infected mice. Moreover, *H. pylori* infection-elevated ASCL1 and AQP5 expression promoted apoptosis and inflammation in GECs. Taken together, the key findings of the present study demonstrate that *H. pylori* infection activated WNT/β-catenin signaling pathway by upregulating ASCL1/AQP5 to induce gastritis.

## Introduction

Gastritis is an acute or chronic, diffuse or localized gastric wall swelling, whose most common symptoms include epigastric pain, heartburn, nausea, and vomiting [1]. Gastritis can be classified into three types, autoimmune, bacterial, and chemical gastritis [2]. Accumulating evidence has reported that *Helicobacter pylori (H. pylori)* infection contributes to the progression of gastritis [3]. *H. pylori*, known as a Gram-negative bacterium, can infect over half of the world’s population [4]. *H. pylori* colonizes the human gastric mucosa and persists for decades, which results in gastritis and eventually progresses to gastric cancer [5]. *H. pylori*, classified as a class I carcinogen by World Health Organization, is a critical factor contributing to gastritis [6]. Over the past decades, the studies about *H. pylori* in gastritis have focused on the etiology, natural history, and prognosis of gastritis [7]. However, the mechanisms of developing *H. pylori*-associated gastritis remain unclear [8].

It is interesting to note that *H. pylori* infection could elevate Aquaporin 5 (AQP5) expression in gastric epithelial cells (GECs), which promotes the development of gastritis [9]. AQP5, a member of AQPs, is essential for regulating water transport in healthy tissues and is related to multiple human cancers, such as gastric cancer [10]. As serpentine membrane proteins, aquaporins (AQPs) promote the movement of water across the biological membrane [11]. Intriguingly, *H. pylori* infection has been documented to enhance AQP5 levels in gastric epithelial cells, while ablation of AQP5 was found to negate *H. pylori-*triggered cell invasive and proliferative properties [9].

Moreover, bioinformatics analysis in the current study manifested that there was a binding relationship between achaete-scute complex-like 1 (ASCL1) and AQP5. ASCL1 belongs to ASCL gene family that is known as ‘achaete-scute complex homolog’ or ‘achaete-scute family basic helix-loop-helix transcription factor’ and mammalian achaete-scute homologues (MASH) [12]. High ASCL1 (also known as MASH1) expression has been suggested in the glandular stomach epithelium, and it may be responsible for the development of neuroendocrine cells in the glandular stomach [13]. In addition, the study by Kim et al. also suggested that ASCL1 may be expressed in endocrine progenitors of the stomach and its loss reduces endocrine cells [14]. Furthermore, AQP5 depletion in colorectal cancer cells could repress the WNT/β-catenin pathway activation [15, 16].

Therefore, the mechanism underlying the regulatory mechanism of *H. pylori* infection may associate with AQP5, ASCL1 or the WNT/β-catenin pathway. In this context, our study was performed to delineate the involvement of AQP5 and ASCL1 in *H. pylori* infection during gastritis.

## Results

### AQP5 was highly expressed in gastric mucosa of *H. pylori*-infected mice

**Fig. 1:**
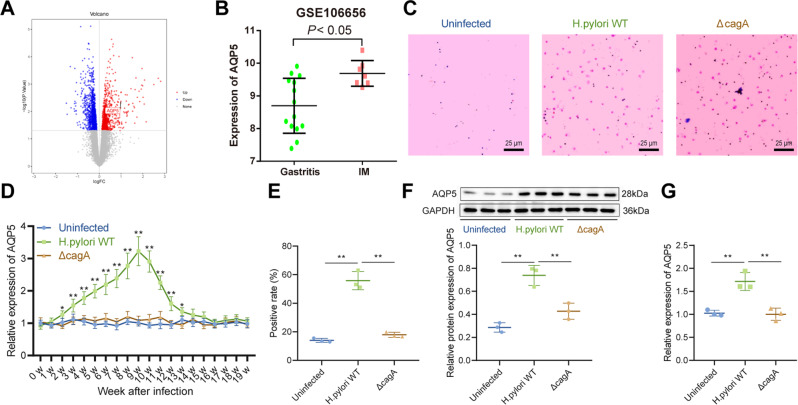
*H. pylori* infection elevates AQP5 expression in gastric mucosa. **A** Volcano map of differentially expressed genes in microarray data GSE106656. Red represents upregulated genes and green represents downregulated genes with |log_2_FC | ≥ 1 and *p* < 0.05. **B**, AQP5 expression in 14 gastritis patients and 7 IM patients in microarray data GSE106656. **C**, *H. pylori* infection in mice detected by Giemsa staining. **D**, AQP5 mRNA level in mouse gastric mucosa determined by RT-qPCR (*n* = 6). **E**, AQP5 protein level in mouse gastric mucosa determined by immunohistochemistry (*n* = 3). **F**, AQP5 protein level in mouse isolated gastric mucosa determined by Western blot analysis (*n* = 3). **G**, AQP5 mRNA level in mouse isolated gastric mucosa determined by RT-qPCR (*n* = 3). *p < 0.05, **p < 0.01. Data were shown as the mean ± standard deviation. Comparisons of data between two groups were analyzed by independent sample *t-*test. One-way ANOVA was applied for the comparison of data among multiple groups. The data between groups at different time points were compared by repeated measures ANOVA.

Initially, bioinformatics analysis was conducted to predict the mechanism underlying *H. pylori* infection-induced gastritis. Through the differential analysis of *H. pylori* infection-induced gastritis-related microarray data GSE106656, we found that AQP5 was significantly overexpressed in intestinal metaplasia (IM) patients compared with gastritis patients (Fig. 1A, B).

To explore the potential role of AQP5 in *H. pylori* infection, mice were infected with *H. pylori* wild type (WT) (11637, *cagA* positive) and Δ*cagA* (11637, *cagA* knockout), which was verified by Giemsa staining (Fig. 1C). RT-qPCR exhibited that AQP5 mRNA level increased in the gastric mucosa of *H. pylori* WT-infected mice and peaked at the 9^th^ week of infection, whilst Δ*cagA* infection did not impact AQP5 levels in mouse gastric mucosa (Fig. 1D).

Immunohistochemistry further displayed that AQP5 protein level elevated in the gastric mucosa of *H. pylori* WT-infected mice, and that AQP5 protein level was not changed in the gastric mucosa of uninfected mice and mice infected with Δ*cagA* (Fig. 1E). In addition, the isolated mouse gastric mucosa was infected with *H. pylori* WT and Δ*cagA*. Compared with uninfected or *ΔcagA*-infected gastric mucosa, AQP5 expression enhanced remarkably in gastric mucosa after *H. pylori* WT infection (Fig. 1F, G).

Thus, AQP5 was upregulated in gastric mucosa of *H. pylori*-infected patients and mice.

### AQP5 expression was upregulated in response to *H. pylori* infection in mouse GECs

**Fig. 2:**
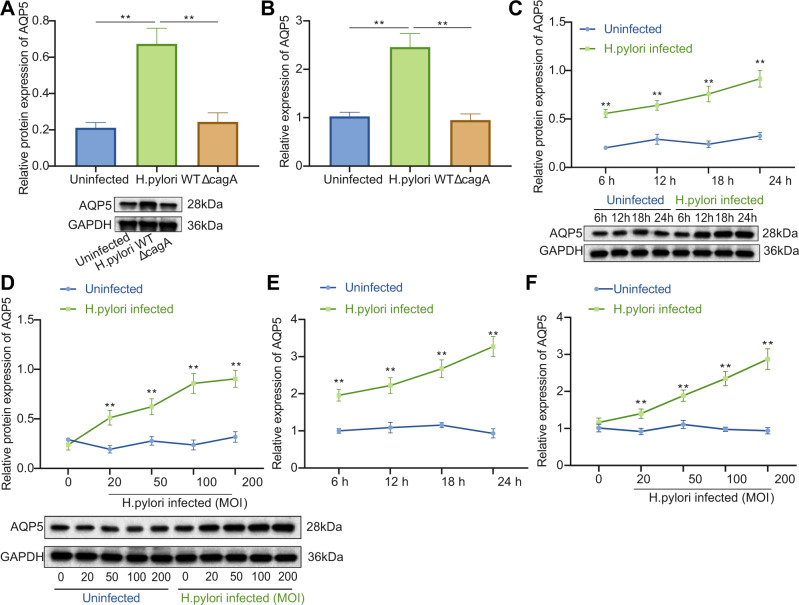
*H. pylori* infection enhances AQP5 expression in mouse GECs. **A** AQP5 protein level in mouse primary GECs measured by Western blot analysis. **B**, AQP5 mRNA level in mouse primary GECs measured by RT-qPCR. **C**, **D**, AQP5 protein level in mouse primary GECs at different time points (**C**) and doses (**D**) measured by Western blot analysis. **E**, **F**, AQP5 mRNA level in mouse primary GECs at different time points (**E**) and doses (**F**) measured by RT-qPCR. *p < 0.05, **p < 0.01. Data were shown as the mean ± standard deviation. Cell experiments were repeated three times independently. Comparisons of data between two groups were analyzed by independent sample *t-*test. One-way ANOVA with Dunnett’s post hoc test was applied for the comparison of data among multiple groups. The data between groups at different time points were compared by repeated measures ANOVA.

GECs are the initial contact sites of bacteria when *H. pylori* infects the host gastric mucosa [17]. Therefore, mouse primary GECs were infected with *H. pylori* (WT and Δ*cagA*) to determine whether GECs were involved in *H. pylori* infection-induced AQP5 high expression. It was found that after *H. pylori* WT infection, AQP5 expression was elevated in primary GECs of mice (Fig. 2A, B) in a time- and dose-dependent manner (Fig. 2C–F). However, Δ*cagA* infection exerted no effects on AQP5 expression (Fig. 2A, B).

Conclusively, *H. pylori* could induce AQP5 expression in primary mouse GECs.

### *H. pylori* induces AQP5 expression by upregulating ASCL1 expression

**Fig. 3:**
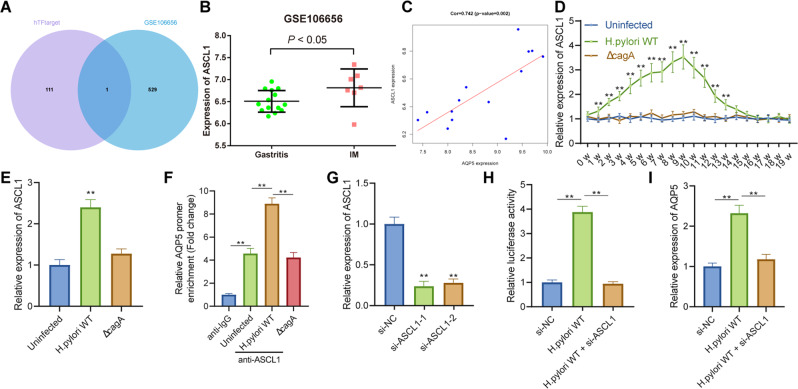
*H. pylori* augments AQP5 expression by upregulating ASCL1. **A** Veen map of the transcription factors binding AQP5 predicted by hTFtarget database and AQP5-related genes in microarray data GSE106656. **B**, ASCL1 expression in 14 gastritis patients and 7 IM patients in microarray data GSE106656. **C** Correlation between ASCL1 and AQP5 expression in microarray data GSE106656. **D** ASCL1 mRNA level in mouse gastric mucosa measured by RT-qPCR (*n* = 6). **E**, ASCL1 mRNA level in mouse primary GECs measured by RT-qPCR. **F** Enrichment of ASCL1 on the AQP5 promoter detected by ChIP assay. **G** The silencing efficiency of si-ASCL1-1 or si-ASCL1-2 as reflected by ASCL1 mRNA level in mouse primary GECs transduced with si-ASCL1-1 or si-ASCL1-2 measured by RT-qPCR. **H** The target relationship between ASCL1 and AQP5 verified by dual luciferase reporter assay. **I** ASCL1 mRNA level in *H. pylori-i*nfected mouse primary GECs transduced with si-ASCL1 measured by RT-qPCR. *p < 0.05, **p < 0.01. Data were shown as the mean ± standard deviation. Cell experiments were repeated three times independently. Comparisons of data between two groups were analyzed by independent sample *t-*test. One-way ANOVA with Dunnett’s post hoc test was applied for the comparison of data among multiple groups. The data between groups at different time points were compared by repeated measures ANOVA.

In order to ascertain the molecular mechanism of high AQP5 expression induced by *H. pylori*, the predicted transcription factors of AQP5 were obtained through hTFtarget database and intersected with the AQP5-related genes obtained in GSE106656. Accordingly, the transcription factor ASCL1 was determined as the candidate gene (Fig. 3A). As per differential analysis results of GSE106656, ASCL1 was highly expressed in IM patients in contrast to gastritis patients (Fig. 3B), and was positively correlated with AQP5 expression (Fig. 3C).

Additionally, ASCL1 mRNA level increased in the gastric mucosa of *H. pylori* WT-infected mice and peaked at the 9^th^ week of infection, but Δ*cagA* infection did not alter AQP5 expression in mouse gastric mucosa (Fig. 3D), which was validated in primary mouse GECs (Fig. 3E). In conclusion, ASCL1 was upregulated in *H. pylori*-infected gastric mucosa and GECs.

To verify the target relationship between ASCL1 and AQP5, the binding sites of transcription factor ASCL1 and AQP5 promoter were predicted by JASPAR website, and the site with the highest score was 5′-GGCACCTGTT-3′ (Table S4). ChIP assay presented that the transcription factor ASCL1 was enriched in the AQP5 promoter, and that *H. pylori* WT infection further facilitated the enrichment of ASCL1 in the AQP5 promoter but *ΔcagA* infection had no effect (Fig. 3F). As reflected by dual luciferase reporter assay and RT-qPCR results, *H. pylori* WT infection augmented the luciferase activity and mRNA level of AQP5 promoter, whereas ASCL1 knockdown abrogated this trend (Fig. 3G-I).

In summary, *H. pylori* induced transcription factor ASCL1 expression, thereby elevating AQP5 transcriptional expression.

### Knockout of AQP5 relieved *H. pylori*-induced gastritis in mice

**Fig. 4:**
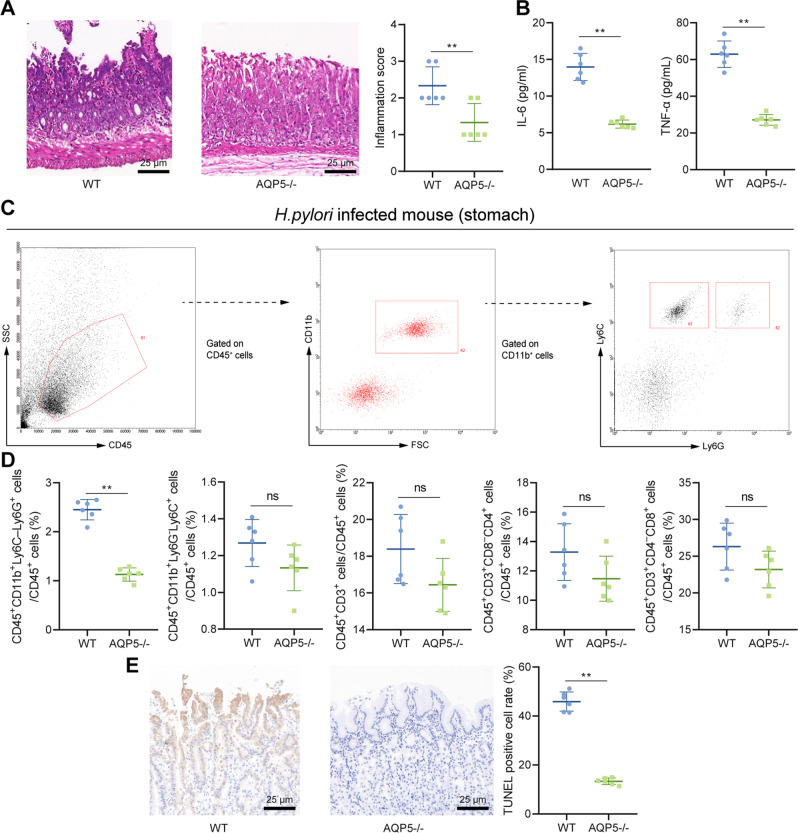
AQP5 knockout attenuates *H. pylori*-induced gastritis in mice. **A** H&E staining and histological score of inflammation in gastric mucosa of mice. **B** Levels of IL-6 and TNF-α in gastric mucosa measured by ELISA. **C** Flow cytometry of isolated neutrophils (CD45^+^CD11b^+^Ly6C^–^Ly6G^+^) and monocytes (CD45^+^CD11b^+^Ly6G^–^Ly6C^+^). **D** The contents of immune cells in gastric mucosa of mice detected by flow cytometry. **E** Quantification of apoptotic cells in gastric mucosa detected by TUNEL assay. *p < 0.05, **p < 0.01, ns not significant. Data were shown as the mean ± standard deviation. *n* = 6 mice/group. Comparisons of data between two groups were analyzed by independent sample *t-*test.

AQP5^−/−^ mice were infected with *H. pylori* WT to assess the role of AQP5 in *H. pylori*-induced gastritis. Moreover, AQP5^−/−^ mice exhibited alleviated gastric mucosal epithelium damage, more orderly arranged proper gland, and reduced inflammatory response and levels of pro-inflammatory cytokines (IL-6 and TNF-α) in gastric mucosa (Fig. 4A, B).

We next moved to explore regulatory effect of AQP5 on inflammatory cell infiltration in mouse gastric mucosa, the proportion of neutrophils (CD45^+^CD11b^+^Ly6C^–^Ly6G^+^), monocytes (CD45^+^CD11b^+^Ly6G^–^Ly6C^+^), CD45^+^CD3^+^T cells, CD45^+^CD3^+^CD8^–^CD4^+^T cells, and CD45^+^CD3^+^CD4^–^CD8^+^T cells in mouse gastric mucosa was measured by flow cytometry. It was revealed that neutrophils were diminished in gastric mucosa of AQP5^−/−^ mice, but there was no change in T cells (Fig. 4C, D). TUNEL assay also displayed that apoptotic cells were reduced in gastric mucosa of AQP5^−/−^ mice (Fig. 4E).

Therefore, AQP5 knockout suppressed neutrophil infiltration and apoptosis in mouse gastric mucosa, thereby alleviating *H. pylori-*induced gastritis.

### *H. pylori* infection activated the Wnt/β-catenin pathway by upregulating AQP5

**Fig. 5:**
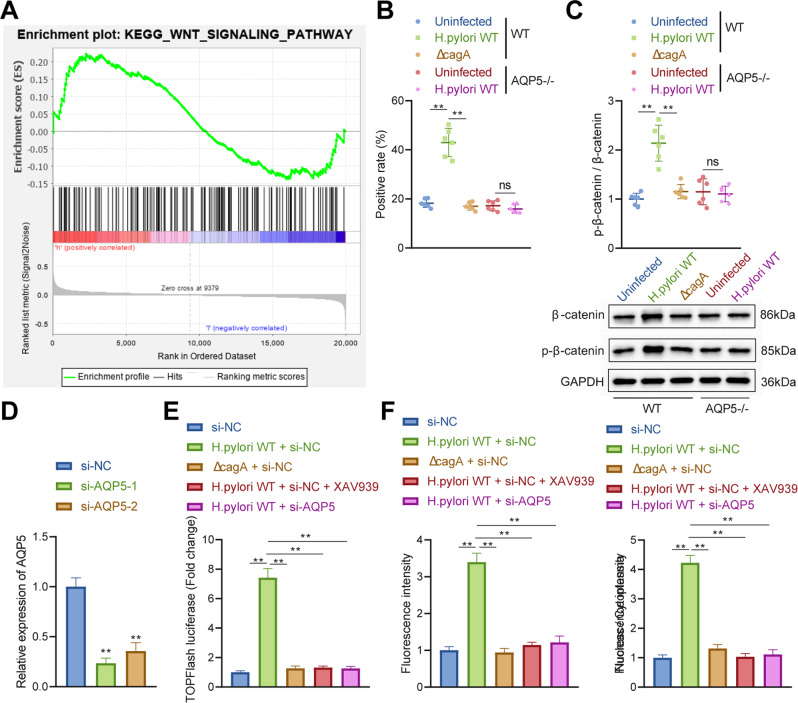
*H. pylori* infection facilitates the activation of Wnt/β-catenin pathway by upregulating AQP5. **A** The differences in enriched pathways between AQP5 high expression and low expression groups analyzed by GSEA. **B** β-catenin expression in gastric mucosa of mice detected by immunohistochemistry (*n* = 6). **C** β-catenin protein level in gastric mucosa of mice detected by Western blot analysis (*n* = 6). **D** AQP5 mRNA level in AGS cells determined by RT-qPCR. **E** The activation of Wnt/β-catenin pathway in primary GECs of mice detected by TOPFlash luciferase reporter assay. **F** β-catenin expression in mouse primary GECs detected by immunofluorescence staining. *p < 0.05, **p < 0.01, ns not significant. Data were shown as the mean ± standard deviation. Cell experiments were repeated three times independently. One-way ANOVA with Dunnett’s post hoc test was applied for the comparison of data among multiple groups.

Gene Set Enrichment Analysis (GSEA) analysis was performed on GSE106656 to further analyze the downstream mechanism of AQP5, which revealed that highly expressed AQP5 was correlated with Wnt/β-catenin pathway (Fig. 5A). Therefore, it can be speculated that *H. pylori* infection could promote AQP5 expression to activate Wnt/β-catenin pathway.

Next, immunohistochemistry and Western blot analysis revealed that *H. pylori* infection augmented β-catenin expression and nuclear localization in gastric mucosa of WT mice, but Δ*cagA* infection did not exert significant effect. Besides, *H. pylori* infection did not affect β-catenin expression and nuclear localization in gastric mucosa of AQP5^−/−^ mice (Fig. 5B, C). Accordingly, *H. pylori* infection activated Wnt/β-catenin pathway by upregulating AQP5 in mouse gastric mucosa.

As displayed in Fig. 5D-F, WT *H. pylori* infection enhanced TOPFlash luciferase activity in primary mouse GECs (Fig. 5E), the β-catenin fluorescence intensity, and nuclear localization ratio (Fig. 5F), whilst no significant difference was found after *ΔcagA* infection. Treatment with XAV939, a heterogeneous inhibitor of WNT/β-catenin pathway, or AQP5 knockdown obviously nullified the effects caused by WT *H. pylori* infection (Fig. 5E, F).

Together, *H. pylori* infection activated Wnt/β-catenin pathway in mouse GECs by elevating AQP5 expression.

### *H. pylori* facilitated apoptosis and inflammatory responses in GECs *via* the ASCL1/AQP5/Wnt/β-catenin axis

Furthermore, the primary GECs were transduced with si-ASCL1 or treated with XAV939 to probe the influences of *H. pylori* in GECs by manipulating ASCL1/AQP5-mediated activation of Wnt/β-catenin pathway.

**Fig. 6:**
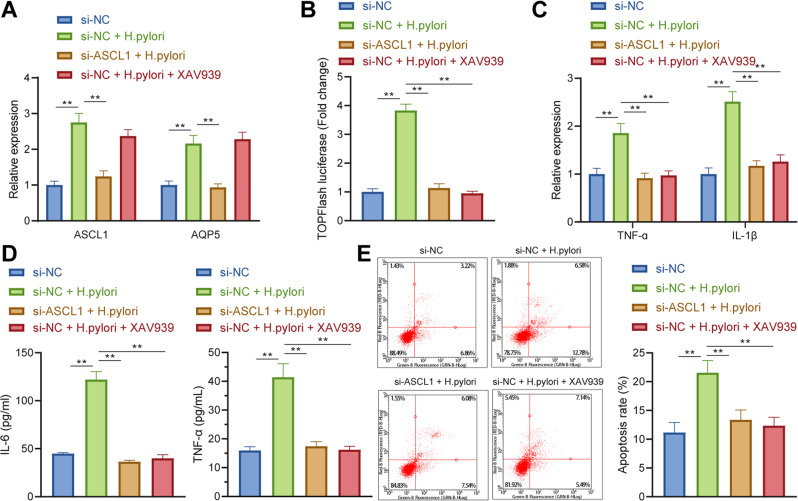
*H. pylori* promotes GEC apoptosis and inflammatory response via ASCL1/AQP5/Wnt/β-catenin axis. **A** mRNA levels of ASCL1 and AQP5 in mouse primary GECs after ASCL1 knockdown or XAV939 treatment determined by RT-qPCR. **B** The activation of Wnt/β-catenin pathway in mouse primary GECs after ASCL1 knockdown or XAV939 treatment detected by TOPFlash luciferase reporter assay. **C** mRNA levels of TNF-α and IL-1β in mouse primary GECs after ASCL1 knockdown or XAV939 treatment determined by RT-qPCR. **D** Protein levels of TNF-α and IL-1β in mouse primary GECs after ASCL1 knockdown or XAV939 treatment determined by ELISA. **E** Apoptosis of mouse primary GECs after ASCL1 knockdown or XAV939 treatment detected by Annexin V/PI staining. *p < 0.05, **p < 0.01. Data were shown as the mean ± standard deviation. Cell experiments were repeated three times independently. One-way ANOVA with Dunnett’s post hoc test was applied for the comparison of data among multiple groups.

RT-qPCR exhibited that silencing of ASCL1 repressed the elevation of ASCL1 and AQP5 by *H. pylori* infection, while XAV939 treatment did not change ASCL1 and AQP5 expression (Fig. 6A). Meanwhile, ASCL1 knockdown or XAV939 annulled *H. pylori*-induced activation of Wnt/β-catenin pathway (Fig. 6B).

Moreover, RT-qPCR and ELISA manifested that TNF-α and IL-1β levels were elevated in GECs following *H. pylori* infection, which were counterweighed by ASCL1 knockdown or XAV939 (Fig. 6C, D). Furthermore, ASCL1 knockdown or XAV939 suppressed *H. pylori*-induced apoptosis of GECs (Fig. 6E).

Cumulatively, *H. pylori* facilitated GEC apoptosis and inflammatory response by activating Wnt/β-catenin pathway via ASCL1/AQP5 axis.

## Discussion

Data obtained in our study elaborated that *H. pylori* infection facilitated inflammatory response and apoptosis of GECs to promote gastritis through the activation of AQP5/ASCL1/WNT/β-catenin axis.

The finding in the present study uncovered that AQP5 was upregulated in gastric mucosa and GECs infected with *H. pylori*. Similarly, a published work has also revealed that AQP5 expression elevates in GECs in gastritis, which is related with the *H. pylori* infection [9]. Moreover, it was found that silencing of AQP5 could restrain levels of inflammatory factor (TNF-α and IL-1β) and apoptosis of GECs in mice with *H. pylori*-related gastritis. GECs are the initial contact sites of bacteria when *H. pylori* infects the host gastric mucosa [17]. During the infection, GECs secrete various cytokines that are implicated in the inflammatory gastric environment after infection with *H. pylori* [4]. A recent study has highlighted that *H. pylori* stimulates inflammatory cytokines, especially IL-1β and TNF-α, to induce chronic gastric inflammation [18]. Exiting literature has also indicated *H. pylori* infection promotes gastric mucosal injury and GEC apoptosis by elevating levels of pro-inflammatory cytokines, leading to gastritis that finally progress to gastric cancer [19, 20]. *H. pylori* infection augments AQP3 expression to accelerate production of proinflammatory cytokines in the pathogenesis of gastric carcinoma [21]. Another study has elucidated that downregulation of AQP5 could inhibit immune cell migration and inflammatory reaction during sepsis [22].

Aquaporins has been studied in the digestive system [23], but their expression and regulation are mainly investigated in the digestive glands and their function are mainly examined in in vitro experiments. AQP5 expression is mainly witnessed in the lower stomach and duodenum of rats, and its effect on water transport mechanism may be conferred there [24]. The study of Matsuzaki et al. [25] has identified that the function of AQP5 in the digestive system was achieved through water transfer in apical membrane, including intercellular secretory canaliculi of secretory cells in the minor salivary glands, pyloric glands, and duodenal glands. Verkman et al. have constructed transgenic mice lacking AQP5 and defective saliva production was observed in response to AQP5 [26], meanwhile, they also found many examples without any obvious phenotypic abnormality in response to tissue-specific expression of an aquaporin. The findings of Ma et al. on aquaporin water channels in gastrointestinal physiology [27] have pointed out that no direct data was witnessed for a role of aquaporins in gastrointestinal physiology, and they suggested that a role for AQP1 in dietary fat processing and AQP4 in colonic fluid absorption.

In the current study, bioinformatics analysis showed that AQP5 was highly expressed in patients with IM of microarray data. In addition, in vitro experiments further confirmed the high expression of AQP5 in gastric mucosal epithelial cells of *H. pylori*-infected mice. Furthermore, in vivo mouse experiments confirmed that compared with WT mice, *H. pylori*-infected AQP5^−/−^ mice exhibited attenuated destruction of gastric mucosal epithelium, more orderly arranged proper gland, and reduced inflammatory response and levels of pro-inflammatory cytokines (IL-6 and TNF-α) in gastric mucosa. After AQP5 knockout, gastric mucositis weakened, gastric mucosal integrity was restored and relieved, and the secretion of proinflammatory factors decreased. These findings suggested that AQP5 had a damaging effect on the gastrointestinal health of mice. The absence of AQP5 promoted the resistance of mice to gastroenteritis. AQP5 played a potential damaging role in the intestinal mucosal barrier and the secretion of intestinal secretory cells and glands. However, the specific impact of AQP5 on the physiological function of gastrointestinal tract remains unknown, which is required to further investigated in the future to offer novel therapies to regulate fluid movement in gastrointestinal diseases.

Moreover, further mechanistic investigation data in this study unraveled that *H. pylori* induced AQP5 expression by upregulating ASCL1 that was highly expressed in gastritis. ASCL1 is implicated in the development of a variety of tumors [28]. However, there existed no reports about the role of ASCL1 in *H. pylori* infection and gastritis. Our finding unveiled that ASCL1 was highly expressed in gastritis and *H. pylori* infected-GECs. In addition, the present study demonstrated that *H. pylori* infection activated WNT/β-catenin pathway in GECs by enhancing AQP5 expression. A prior study has revealed that AQP5 knockdown could reduce expression of Wnt1 and β-catenin [16], which is consistent with our findings. Wnt/β-catenin pathway, also known as typical Wnt pathway, is of great significance for embryonic development and homeostasis of adult tissues [29]. It has been validated that *H. pylori* infection activates Wnt/β-Catenin pathway to induce the inflammatory process of gastric epithelial mucosa, as well as the progression of gastric cancer cells [18]. Another study has also indicated that the inhibition of Wnt/β-catenin pathway by Jianpiyiqi formula could relieve chronic atrophic gastritis in rats [30]. These evidences support that *H. pylori* infection triggered the inflammatory response and apoptosis of GECs to induce the occurrence of gastritis by activating Wnt/β-catenin pathway via upregulation of ASCL1 and AQP5.

**Fig. 7:**
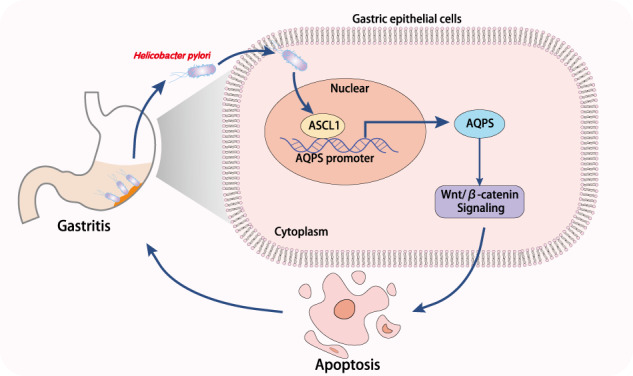
Molecular mechanisms of *H. pylori* infection involved in the occurrence of gastritis. *H. pylori* infection induces AQP5 expression by promoting ASCL1 expression to activate the Wnt/β-catenin pathway, which led to the inflammation and apoptosis of GECs in gastritis.

To sum up, our study supported the notion that *H. pylori* infection activated AQP5 expression by promoting ASCL1 expression to activate the Wnt/β-catenin pathway, which led to the inflammation and apoptosis of GECs in gastritis (Fig. 7). Our findings provided novel insights for the development of therapeutic strategies for inhibiting the occurrence of gastritis induced by *H. pylori* infection. However, the translation of the finding into clinical scenario may be limited by the lack of verification of the expression and distribution of AQP5 and ASCL1 in clinical samples of gastritis and IM, which should be further studied in future investigations.

## Materials and methods

### Ethics statement

All animal experiments were performed with review and approval from the Animal Ethical and Experimental Committee of The First Affiliated Hospital of Nanchang University.

### Bioinformatics analysis

Expression of AQPs-related genes in was predicted in microarray data GSE106656 from Gene Expression Omnibus database (14 patients with gastritis and 7 patients with IM infected with *H. pylori*). The Pearson correlation analysis was adopted to screen out the AQP5-related genes in patients with gastritis with |r | > 0.5 and *p* < 0.05, and GSEA was utilized to reveal the differences in enrichment pathways between AQP5 high expression and low expression. Transcription factors binding to gene promoters were predicted using hTFtarget database. The overlapped genelist in the comparison groups was found with jvenn tool.

### Establishment of AQP5^−/−^ mice

C57BL/6 mice (Beijing Vital River Laboratory Animal Technology Co., Ltd., Beijing, China) were subjected to CRISPR-Pro gene knockout technique. The corresponding gRNA plasmid of mouse AQP5 target gene was designed and constructed. After transcription into RNA in vitro, F0 heterozygous mice with positive sequencing identification were obtained by prokaryotic microinjection with Cas9 mRNA. Next, F0 generation heterozygous mice were mated with WT mice to obtain F1 generation heterozygous mice with positive PCR and sequencing identification. F1 generation mice with the same genotype from the same F0 generation mice were selected and matched with each other after sexual maturity to obtain F2 generation mice. The F2 generation mice were identified by PCR and sequencing to obtain AQP5^−/−^ mice. In addition, WT mice in the same cage were used as control.

### *H. pylori* culture and in vitro infection

*H*. *pylori* NCTC 11637 (*cagA*-positive) (43504, American Type Culture Collection, Manassas, VA) and *cagA*-KO mutant *H*. *pylori* NCTC 11637 (*ΔcagA*) were grown in brain-heart infusion plates supplemented with 10% rabbit blood at 37 °C under microaerophilic conditions (10% CO_2_, 5% O_2_, 85% N_2_). The density of bacteria was assessed by measuring its absorbance at 600 nm with 1 OD600 = 1 × 10^9^ CFU/mL.

The bacteria were collected and resuspended in serum-free Roswell Park Memorial Institute (RPMI) 1640. For infecting cells, after counting, cells were infected with bacterial suspension (MOI = 100) at a ratio of 100 (bacteria): 1 (cells) for 24 h. For the infected isolated tissues, the gastric antral mucosa of fresh uninfected patients or mice was ground into single-cell suspension, and then infected in bacterial suspension (MOI = 100) for 24 h.

### Constructions of *H. pylori-*infected gastritis mouse models

A total of 30 female C57BL/6 WT mice were fed in specific pathogen free animal laboratory under controlled conditions of 60–65% humidity, 22–25 °C temperature, 12-h light/dark cycle, and free access to food and water. One week of acclimatization was allowed prior to the experiment. Six mice were randomly assigned to each group. The modeled mice (aged 8–10 weeks, weighing 22–24 g) were administered with 3 × 10^8^ CFU *H. pylori* (0.4 mL normal saline) by gavage every other day, twice a day, and ended within 1 h.

The infection status of *H. pylori* in mice was evaluated by RT-qPCR, rapid urease test (Shandong Biomedia Laboratories Co., Ltd., Jinan, China), and Giemsa staining (380050-g, Beijing CellChip Biotechnology Co., Ltd., Beijing, China). Mice were divided into the following groups: uninfected (WT mice), *H. pylori* (WT mice), *H. pyloriΔcagA* (WT mice), and *H. pylori* (AQP5^−/−^ mice).

### Evaluation of bacterial colonization

As per a previous study [31], the DNA of mouse gastric mucosa was extracted with QIAamp DNA Mini Kit (Qiagen company, Hilden, Germany). *H. pylori* colonization was quantified using *H. pylori* specific 16 *S* rDNA with specific primers and probes using TaqMan method. β2-microglobulin DNA (mouse) in the same tissues served as the internal reference. *H. pylori* density in the sample was represented as the number of bacterial genomes per nanogram of host genomic DNA. Primer and probe information are shown in Table S1.

With respect to the probes, a FAM fluorescent reporter was coupled to the 5′ end, and a TAMRA quencher to the 3′ end.

### Hematoxylin-eosin (H&E) staining and immunohistochemical staining

The mice were euthanized by anesthetic overdose. The larger gastric curvature was cut, followed by fixation and paraffin embedding. The paraffin-embedded gastric mucosa was cut into 3 µm sections using a semi-automatic slicer (Leica microsystems GmbH, Wetzlar, Germany), which were treated with H&E staining and immunohistochemical staining.

H&E Kit (C0105, Beyotime, Shanghai, China) was applied for staining. In brief, subsequent to dewaxing in xylene and hydration with absolute ethanol, the sections underwent 5–10 min of hematoxylin staining. The excess dye was washed by immersing in running water for 10 min, followed by staining with eosin for 30 s–2 min and immersion in ethanol of 70%, 80%, and 90% and absolute ethanol for 10 s, respectively. The sections were cleared with xylene for 5 min, and with fresh xylene for another 5 min. Subsequent to neutral balsam sealing, the sections received observation and photographing under the inverted microscope (IX73, Olympus, Tokyo, Japan). The film was evaluated by two pathologists using double-blind method according to the standard, 0 = normal, 1 = mild, 2 = moderate, and 3 = significant to obtain the average score [32].

In terms of immunohistochemical detection, after dewaxing and hydration, the activity of endogenous peroxidase was blocked by culturing the sections with 3% hydrogen peroxide. The slides underwent 30-min boiling in 10 mM sodium citrate (pH 6.0) and 15-min blocking in 10% normal goat serum, followed by incubation with antibodies to AQP5 (PA5-97290, 1:100, Invitrogen, Carlsbad, CA) or β-catenin (Ab32572, 1:100, Abcam, Cambridge, UK) in a wet chamber at 4 °C overnigh. The next day, the slides underwent 1-h incubation with the secondary antibody (Ab6721, 1:500, Abcam) at ambient temperature. The immune reactivity was evaluated with diaminobenzidine (DAB) Kit (Invitrogen).

### TUNEL assay

Apoptosis in mouse gastric mucosa was measured following the manuals of TUNEL Kit (C1098, Beyotime). After inactivation of endogenous peroxidase using 3% H_2_O_2_, the sections received 60-min culture with 50 μL TUNEL avoiding light exposure at 37 °C and 30-min incubation with 50 μL Streptavidin-HRP working solution. The sections were stained with 0.2–0.5 mL DAB based on the size of tissue block at ambient temperature for 5 min. After sealing, five different visual fields were chosen using an inverted microscope (IX73, Olympus), followed by observation and photographing. The total number of cells and the number of apoptotic cells were counted respectively before calculation of apoptosis rate.

### Flow cytometric sorting

The gastric mucosal tissue was cut into several small squares, ground into single-cell suspension, centrifuged, moistened with PBS, and stained with cell surface marker specific or isotype control antibody. After filtration with nylon mesh, it was analyzed by multicolor flow cytometry on FACSCanto II (BD Biosciences, San Jose, CA). The data were analyzed by FlowJo (Tree Star Inc.) or FACSDiva software (BD Biosciences).

The antibodies (BioLegend, San Diego, CA) were as follows: anti-mouse CD45-PE-Cy7 (stock no. 103113), anti-mouse CD11b-PerCP-Cy5.5 (stock no. 101227), anti-mouse Ly6G-FITC (stock no. 127605), anti-mouse Ly6C-PE (stock no. 128007), anti-mouse CD3-APC (stock no. 100235), anti-mouse CD8-PerCP-Cy5.5 (stock no. 100733), and anti-mouse CD4-PE (stock no. 100407).

### Enzyme-linked immunosorbent assay (ELISA)

For gastric mucosal tissue, 1 mL sterile protein extraction reagent was homogenized and centrifuged (13,000 *g*, 4 °C, 10 min) to harvest the supernatant for ELISA. The supernatant of gastric mucosal epithelial cell line underwent centrifugation (2000 *g*, 4 °C, 10 min) to discard precipitate. The content of IL-1β and TNF-α was assessed by ELISA kits (Abcam), including human-IL-1β (ab214025), mouse-IL-1β (ab197742), human-TNF-α (ab181421), and mouse-TNF-α (ab208348).

### Cell culture and transduction

The fresh normal mouse gastric mucosal tissues (*H. pylori* was negative) were obtained. The tissues were washed with RPMI 1640 (21875034, Gibco, Carlsbad, California) encompassing 1% fetal bovine serum (FBS) three times, and cut into blocks, which were attained in the RPMI 1640 supplemented with collagenase IV (1 mg/mL, 17104019, Gibco) and DNase I (10 mg/mL, 11284932001, Sigma-Aldrich, St. Louis, MO). Next, cells were dissociated with gentle machinery, and rotated at 37 °C for 0.5–1 h. The cells were filtered with 70-μm cell filter (BD Labware), and purified using anti-CD326 (EpCAM) magnetic beads (Miltenyi Biotec) in the MACS column purification system [33]. When cell viability was > 90% (MTT method) and purity was > 95% (PAS staining, G1360, Solarbio, Beijing, China), they could be identified as primary gastric epithelial cells (GECs).

Mouse primary GECs were cultured in RPMI 1640 medium encompassing 10% FBS (10099141, Gibco) and 1% penicillin streptomycin (15070063, Gibco) with 5% CO_2_ at 37 °C.

Cell transduction was conducted using Lipofectamine 2000 (11668019, Invitrogen) with small interfering RNA (siRNA/si)-negative control (NC), si-AQP5-1, si-AQP5-2, si-ASCL1-1, and si-ASCL1-2. siRNA sequences (Table S2) were designed using BLOCK-iT™ RNAi Designer and synthetized by GenePharma (Shanghai, China). Cells were transduced.

### RT-qPCR

Trizol (16096020, Invitrogen) was employed for total RNA isolation from tissues. RNA was generated into cDNA using the Reverse Transcription Kit (RR047A, Takara, Tokyo, Japan). RT-qPCR was implemented on the real-time fluorescence quantitative PCR instrument (ABI 7500, Applied Biosystems, Carlsbad, CA). All primers are shown in Table S3 and designed using NCBI, and the 2^−ΔΔCt^ method was used to quantify relative expression levels of genes as normalized to GAPDH.

### Western blot analysis

The total protein was extracted from mouse gastric mucosa tissues or cells using radio immunoprecipitation assay lysis (Beyotime). The protein concentration was estimated using BCA Kit (20201ES76, Yeasen Company, Shanghai, China). Subsequent to separation using sodium dodecyl sulfate-polyacrylamide gel electrophoresis gels, the protein was electrotransferred onto a polyvinylidene fluoride membrane (IPVH85R, Millipore, Germany). Subsequent to 1-h 5% bovine serum albumin blocking, the membrane received overnight probing at 4 °C with the primary antibodies to AQP5 (PA5-97290, 1:1000, Invitrogen), β-catenin (Ab32572, 1:1000, Abcam), p-β-catenin (Ab246504, 1:1000, Abcam), and GAPDH (ab8245, 1:5000, Abcam) before 1-h re-probing incubated with HRP-labeled goat anti-rabbit IgG (ab6721, 1:5000, Abcam) or goat anti-mouse IgG (ab6789, 1:5000, Abcam) at ambient temperature. The membrane was developed with luminescent liquid. The protein was qualified using ImageJ software (National Institutes of Health) as normalized to internal reference (GAPDH).

### Cellular immunofluorescence

Cells were fixed with 4% paraformaldehyde before membrane rupture with 0.1% Triton X-100. Subsequent to blocking with 10% normal donkey serum, the membrane received overnight probing at 4 °C with rabbit anti-β-catenin (Ab32572, 1:100, Abcam) before 1-h re-probing with the secondary antibody secAlexa Fluor594-coupled goat anti-rabbit IgG (ab150080, 1:200, Abcam) at ambient temperature. Subsequent to DAPI (C1006, Beyotime) staining, the cells were observed under the fluorescence microscope (BX63, Olympus), and analyzed by ImageJ software.

### Flow cytometric apoptosis detection

The apoptosis was evaluated by Annexin V-FITC/PI double staining. Cells with 2 × 10^5^ cells/well were inoculated on 6-well plates, followed by trypsinization. Cells were collected in 15 mL centrifuge tube for centrifugation at 800 *g* to remove supernatant. In the light of the apoptosis detection kit (556547, BD Bioscience), the precipitates were resuspended in 500 μL binding buffer and mixed with 5 μL FITC and 5 μL PI in the dark for 15 min before apoptosis analysis in a flow cytometer (BD FACSCalibur).

### Chromatin immuno-precipitation (ChIP) assay

The EZ-Magna ChIP TMA Kit (Millipore) was adopted for ChIP assay to measure the enrichment of ASCL1 the AQP5 promotor. Logarithmically growing cells underwent 10-min incubation with 1% formaldehyde, and crosslinking was terminated by 125 mM glycine. The 200–1000 BP chromatin fragments were yielded by ultrasonic lysis. Cells received 10-min centrifugation at 4 °C and 14,000 *g* to aspirate supernatant. Cell supernatant (100 μL, DNA fragment) was added with 900 μL ChIP Dilution Buffer and 20 μL 50 × PIC, and then added with 60 μL ProteinA Agarose/Salmon Sperm DNA, mixing at 4 °C for 1 h and standing at 4 °C for 10 min. Cells were centrifuged at 700 *g* for 1 min to collect supernatant. The 20 μL supernatant served as Input. In the experimental group, the supernatant was added with 1 μL ASCL1 rabbit antibody (43666, CST, Beverly, MA) and the NC group was added with 1 μL rabbit antibody IgG (ab172730, Abcam). Each tube was added with 60 μL ProteinA Agarose/Salmon Sperm DNA at 4 °C for 2 h. After standing for 10 min, cells were centrifuged at 700 *g* for 1 min for removal of supernatant. The precipitate was washed with l mL low salt buffer, high salt buffer, LiCl solution, and TE (twice). Each tube was washed with 250 μL ChIP Wash Buffer two times, and de-crosslinking with 20 μL 5 M NaCl to recycle DNA. The enriched chromatin fragments were detected by fluorescence quantitative PCR (AQP5 promoter primer F: 5′-TGCCTGACAAACTGACAG-3′; R: 5′-TGGTGCTAAGCTAGGGGGAA-3′).

### TOPFlash luciferase reporter assay

The activity of Wnt/β-catenin pathway was detected by TCF Reporter Plasmid Kit (17-285, Millipore). The mouse primary GECs were transduced with pTopFlash (TCF reporter plasmid) or pFopFlash (mutant, inactive TCF binding site) plasmids, and pSV40-Renilla (Promega, E6911) as internal reference. After transfection for 48 h, cells were centrifuged at 12000 g for 1 min to collect supernatant. The Dual-Luciferase® Reporter Assay System (E1910, Promega) was adopted to detect luciferase activity. The ratio of firefly luciferase to renilla luciferase was used as the relative activity of luciferase.

### Dual luciferase reporter assay

The full-length dual luciferase reporter plasmid encompassing AQP5 promoter was constructed. The dual luciferase reporter plasmid was transfected into mouse primary GECs. The GECs were lysed 48 h after transfection and centrifuged at 12,000 *g* for 1 min to collect supernatant. The Dual-Luciferase® Reporter Assay System (E1910, Promega, Madison, WI) was adopted to detect luciferase activity as normalized to renilla luciferase activity.

### Statistical analysis

All data were analyzed by graphPad Prism 7.0. The *p* value was determined by two tailed *t*-test on independent samples. The measurement data were presented as mean ± standard deviation. Comparisons of data between two groups were analyzed by independent sample *t-*test, and comparison of data among multiple groups was tested by one-way analysis of variance (ANOVA). The data between groups at different time points were compared by repeated measures ANOVA. *p* < 0.05 was statistically significant.

## Supplementary information

Supplementary Tables

## Data Availability

The datasets generated and/or analysed during the current study are available in the manuscript and supplementary materials.
