# Supplementary material for: Helicobacter pylori infection activates Wnt/β-catenin pathway to promote the occurrence of gastritis by upregulating ASCL1 and AQP5
Source: Cell Death Discov. 2022 May 10;8:257. doi: 10.1038/s41420-022-01026-0 (PMC9090998; doi:10.1038/s41420-022-01026-0)
Supplement: Supplementary file 1 — Supplementary Tables [file 41420_2022_1026_MOESM1_ESM.docx]

**Table S1 Primer and probe sequences**

| Name | Sequences (5'-3') |
| --- | --- |
| *H. pylori* 16s rDNA | Forward: TTTGTTAGAGAAGATAATGACGGTATCTAAC |
|  | Reverse: CATAGGATTTCACACCTGACTGACTATC |
|  | Probe: CGTGCCAGCAGCCGCGGT |
| Mouse β2-microglobulin | Forward: CCTGCAGAGTTAAGCATGCCAG |
|  | Reverse: TGCTTGATCACATGTCTCGATCC |
|  | Probe: TGGCCGAGCCCAAGACCGTCTAC |

Note: For the probes, a FAM fluorescent reporter is coupled to the 5' end, and a TAMRA quencher is coupled to the 3' end.

**Table S2 siRNA sequences**

| Plasmids | Sequences (5'-3') |
| --- | --- |
| si-NC | GATCGTACTCACATCCACACT |
| si-AQP5-1 | TGGCCACCCTCATCTTCGTCTTCTT |
| si-AQP5-2 | GCCGTGGTGGTGGAGTTAATCTTGA |
| si-ASCL1-1 | CCCAACTACTCCAACGACTTGAACT |
| si-ASCL1-2 | CAGAGGAACAAGAGCTGCTGGACTT |

Note: si-, small interfering RNA; AQP5, aquaporin 5; ASCL1, achaete-scute complex-like 1.

**Table S3 Primer sequences for RT-qPCR**

| Genes | Sequences (5'-3') |
| --- | --- |
| AQP5 | Forward: CGCTCAGCAACAACACAACA |
|  | Reverse: CAGCCGGTGAAGTAGATCCC |
| ASCL1 | Forward: ACTTGAACTCTATGGCGGGT |
|  | Reverse: GAACCAGTTGGTAAAGTCCAGCA |
| GAPDH | Forward: AAGGTCATCCCAGAGCTGAA |
|  | Reverse: CTGCTTCACCACCTTCTTGA |
| TNF-α | Forward: GCTGAGGTCAATCTGCCCAA |
|  | Reverse: GGGGCTCTGAGGAGTAGACA |
| IL-1β | Forward: GTCGCTCAGGGTCACAAGAA |
|  | Reverse: GTGCTGCCTAATGTCCCCTT |

Note: AQP5, aquaporin 5; ASCL1, achaete-scute complex-like 1; GAPDH, glyceraldehyde-3-phosphate dehydrogenase; TNF-α, tumor necrosis factor-α; IL-1β, interleukin-1β.

**Table S4 Binding sites of ASCL1 and AQP5 promoter predicted by JASPAR website**

| Name | Score | Relative score | Target | Start | End | Strand | Predicted sequence |  |
| --- | --- | --- | --- | --- | --- | --- | --- | --- |
| ASCL1 | 10.5542 | 0.934195536 | AQP5 | 195 | 204 | + | ggcacctgtt |  |
| ASCL1 | 10.1407 | 0.872000177 | AQP5 | 1552 | 1564 | + | ggaggagctggac |  |
| ASCL1 | 9.65633 | 0.863863262 | AQP5 | 20 | 32 | + | ggaggagctgctc |  |
| ASCL1 | 8.15411 | 0.838628789 | AQP5 | 1797 | 1809 | + | ccggcggctgccc |  |
| ASCL1 | 8.12588 | 0.885003451 | AQP5 | 1867 | 1876 | + | cgcagcggcc |  |
| ASCL1 | 7.97163 | 0.881878767 | AQP5 | 1799 | 1808 | + | ggcggctgcc |  |
| ASCL1 | 7.7997 | 0.832675356 | AQP5 | 193 | 205 | + | gaggcacctgttc |  |
| ASCL1 | 7.57404 | 0.82888474 | AQP5 | 507 | 519 | + | gaggcagcaggtc |  |
| ASCL1 | 6.78042 | 0.815553456 | AQP5 | 1865 | 1877 | + | cccgcagcggcct |  |
| ASCL1 | 6.57271 | 0.853540487 | AQP5 | 509 | 518 | + | ggcagcaggt |  |
| ASCL1 | 5.92427 | 0.840404976 | AQP5 | 455 | 464 | + | tccacatgcc |  |
| ASCL1 | 5.36444 | 0.829064318 | AQP5 | 1778 | 1787 | + | gtcacgtggc |  |
| ASCL1 | 5.29768 | 0.827712 | AQP5 | 22 | 31 | + | aggagctgct |  |
| ASCL1 | 4.97425 | 0.821160062 | AQP5 | 357 | 366 | + | cacacatggg |  |
| ASCL1 | 4.85366 | 0.818717391 | AQP5 | 1925 | 1934 | + | gccccctgcc |  |
| ASCL1 | 4.73358 | 0.81628491 | AQP5 | 621 | 630 | + | accacatgta |  |
| ASCL1 | 4.61162 | 0.813814246 | AQP5 | 1275 | 1284 | + | cccagctacc |  |
| ASCL1 | 4.50915 | 0.811738536 | AQP5 | 382 | 391 | + | tgcagctcct |  |
